# The Association of Patient Factors, Digital Access, and Online Behavior on Sustained Patient Portal Use: A Prospective Cohort of Enrolled Users

**DOI:** 10.2196/jmir.7895

**Published:** 2017-10-17

**Authors:** Susan S Woods, Christopher W Forsberg, Erin C Schwartz, Kim M Nazi, Judith H Hibbard, Thomas K Houston, Martha Gerrity

**Affiliations:** ^1^ VA Maine Healthcare System Augusta, ME United States; ^2^ VA Portland Health Care System Center to Improve Veteran Involvement in Care Portland, OR United States; ^3^ Connected Care Office Veterans Health Administration, U.S. Department of Veterans Affairs Washington, DC United States; ^4^ Health Policy Research Group University of Oregon Eugene, OR United States; ^5^ Bedford VA Medical Center Center for Healthcare Organization and Implementation Research Bedford, MA United States

**Keywords:** patient portal, personal health record, Internet, broadband, digital inclusion, social determinants

## Abstract

**Background:**

As electronic health records and computerized workflows expand, there are unprecedented opportunities to digitally connect with patients using secure portals. To realize the value of patient portals, initial reach across populations will need to be demonstrated, as well as sustained usage over time.

**Objective:**

The study aim was to identify patient factors associated with short-term and long-term portal usage after patients registered to access all portal functions.

**Methods:**

We prospectively followed a cohort of patients at a large Department of Veterans Affairs (VA) health care facility who recently completed identity proofing to use the VA patient portal. Information collected at baseline encompassed patient factors potentially associated with portal usage, including: demographics, Internet access and use, health literacy, patient activation, and self-reported health conditions. The primary outcome was the frequency of portal log-ins during 6-month and 18-month time intervals after study enrollment.

**Results:**

A total of 270 study participants were followed prospectively. Almost all participants (260/268, 97.0%) reported going online, typically at home (248/268, 92.5%). At 6 months, 84.1% (227/270) of participants had visited the portal, with some variation in usage across demographic and health-related subgroups. There were no significant differences in portal log-ins by age, gender, education, marital status, race/ethnicity, distance to a VA facility, or patient activation measure. Significantly higher portal usage was seen among participants using high-speed broadband at home, greater self-reported ability using the Internet, and routinely going online. By 18 months, 91% participants had logged in to the portal, and no significant associations were found between usage and demographics, health status, or patient activation. When examining portal activity between 6 and 18 months, patients who were infrequent or high portal users remained in those categories, respectively.

**Conclusions:**

Short-term and long-term portal usage was associated with having broadband at home, high self-rated ability when using the Internet, and overall online behavior. Digital inclusion, or ready access to the Internet and digital skills, appears to be a social determinant in patient exposure to portal services.

## Introduction

Hopes for personal health records (PHRs) and patient portals run high. The spread of these technologies has been propelled by the US Electronic Health Record Incentive Program and Meaningful Use [1], and consumers’ desire to access their health information, email their providers, and request appointments online [2]. As a result, patients and health systems alike increasingly view digital health services as important means to enhance patient access, drive self-care, and improve the care experience [3,4].

As access to broadband Internet grows and citizens increasingly connect to the Internet using mobile devices, there are unprecedented opportunities to expand remote patient services. While the digital divide in the United States narrows, some gaps are likely to persist. Internet use among US adults rose from 64% in 2005 to 84% in 2015, with 74% of adults with low incomes and 78% of rural residents currently online [5]. However, lower use continues among adults with less than a high school education (66%), and those aged 65 years and older (58%) [5]. Disparities have also been seen when comparing patients who do and do not register for a patient portal. Older patients, those with lower levels of education or income, and African-Americans and Hispanics appear less likely to register for portals [6-9]. As health information technology gains sophistication and health systems offer more virtual services, these demographic differences can impact health equity and outcomes.

Evidence on the impact of patient portal use is mixed, depending upon which functions are available and the measures that are examined. Most studies show that portals offer convenience and enhanced patient satisfaction, with users feeling more in control of their care [10,11]. Patients who use secure emails, refill medications, and access their clinical notes and test results may improve self-care and increase adherence to treatments [12-16]. In one study of portal use by patients with diabetes and hypertension, using a secure email and refilling medications online were associated with improved outcomes [17]. Overall, portals show great promise as a key adjunct to, or at times a substitute for, traditional care and communication.

Studying the use of patient portals has been challenging. Measures of portal use vary across studies, and differing patient populations tend to be examined. Patients need to be aware of portal availability, register or enroll, initially sign in online, and ultimately have the capacity to use the portal as their needs arise. Portal registrants, a group frequently presented in studies, may not accurately reflect those using a portal. Additionally, the value of portals to patients and health systems is more complex than measuring usage. However, the repeated and sustained use of portals may serve as a proxy for benefit. Understanding the value of portals, therefore, must at least demonstrate initial reach across a patient population and show sustained usage over time.

Although a digital divide with broadband Internet does exist, adults who are online and have a chronic condition are more likely than other online adults to search for health information, read online reviews about medications and treatments, or use online peer support [18,19]. What is less clear is the degree to which disparities exist among patients registered for a portal who log in more often compared to those with less frequent portal use. In one study, patients refilling medications online were found to have fewer prescription interruptions, and this finding persisted across all racial and ethnic subgroups [20]. Conversely, racial disparities in portal use were found among patients with human immunodeficiency virus, with non-white participants having lower portal use to monitor their treatment outcomes [21].

Studies examining portal usage have largely been cross-sectional or evaluated matched cohorts. We conducted an exploratory study, prospectively following patients newly enrolled to use a portal, and explored factors associated with portal usage over time. The aim of the study was to identify patient factors associated with portal usage at 6 months and 18 months after initial enrollment (among those completing verification in person). We sought to describe short-term and long-term portal usage, and to examine Internet-related or digital divide issues among patients who visited the portal less frequently compared to those who did so more often.

## Methods

### Design and Setting

Our study was conducted at a large Department of Veterans Affairs (VA) health care facility, where there is a requirement for a patient to complete in-person identity proofing in order to access all portal functions. While this step could prove cumbersome for some, it offered a natural opportunity to examine patients who presumably had some Internet access and were motivated to register for full portal access. We prospectively followed a cohort of VA patients who completed the in-person identity proofing process to fully access the VA’s secure portal, My Health *e* Vet (MHV). At the time of the study, patients who verified their identity could use MHV to refill medications, securely email their providers, receive tailored wellness reminders, view appointments, access laboratory test results through the *Blue Button* feature, and search the VA health education library [22].

Patients eligible for the study received care at the VA Portland Health Care System (VAPHCS) and completed identity proofing for MHV. Each VA facility supports staff that assist Veterans in completing the process. At the study site, the MHV office was adjacent to the Outpatient Pharmacy located in the main lobby of the medical center. MHV staff informed patients about the study by handing them an informational flyer. When a patient was interested in the study, a “warm hand-off” was completed: MHV staff contacted research staff by phone or instant messaging, who immediately met with the patient. The study coordinator (ES) consented and enrolled all subjects and conducted a health literacy assessment. All baseline survey questions were completed on paper at the time of enrollment or within 30 days, and returned by mail in a prestamped envelope. Follow-up surveys were conducted at 6 months to collect patient-reported portal usage. Participants were emailed a link to the survey, with two reminders sent. Due to the MHV staff’s busy workday, we were unable to collect data on all patients who were informed about the study. Participants received US $20 for the baseline and US $30 for follow-up surveys. The study received approval from the VAPHCS Institutional Review Board.

### Measures

The primary outcome measure was the frequency of portal log-ins during 6-month and 18-month time intervals after study enrollment. Log-in data were obtained on all study subjects from the VA’s national Connected Health Office, using MHV Activity Reports. These data were an objective measure of portal use, and listed the total number of successful and unsuccessful (incomplete) MHV log-ins for each study subject.

The study collected information on factors potentially associated with portal usage. Baseline patient measures included: demographics; distance to a VA facility; and technology access and use, including type and location of Internet connection, comfort with computers, and regular online activity (eg, emailing, shopping, social networking, and searching for health information). To assess patients’ overall use of the Internet, a composite *Internet Use Behavior* measure was created that included 11 online activities: accessing the Internet; email; general search; health search; shopping; banking; geolocation; visiting any social network site; registration on any site; posting on any site; and using Facebook, MySpace, or LinkedIn. High Internet use represented going online for at least 7 activities. Health literacy was approximated using the Short Form Functional Health Literacy Assessment (S-TOFHLA) [23]. Level of patient engagement in their health and health care was measured using the Patient Activation Measure (PAM), which is a validated, 13-item instrument [24]. Subjects were asked to self-report their health status and presence of diabetes, hypertension, heart disease, arthritis, depression, tobacco use, asthma, or chronic pulmonary disease. Questions also solicited beliefs about PHRs, how subjects learned about MHV, and prior training on the use of MHV. Subjects were asked about their expectations for using MHV and its available functions.

### Analysis

Baseline characteristics and outcomes were described using frequencies and percentages for the categorical outcomes. Due to the nonnormal distribution of log-ins over time, portal usage was categorized into 4 distinct categories at each time interval. For 6 months, categories included: 0 or 1 log-in, 2 to 5 log-ins, 6 to 11 log-ins, and 12 or more log-ins. Similarly, at 18 months the categories included: 0 to 2 log-ins, 3 to 17 log-ins, 18 to 35 log-ins, and 36 or more log-ins. These 4 categories of log-ins approximately corresponded to portal use frequencies of never/rare use, less than monthly, once or twice per month, and more than twice per month, respectively. To examine the association between frequency of patient portal use and individual patient characteristics, perceptions, and self-reported behavior, we used univariate Chi-square tests. The associations examined frequency of log-ins during the 6-month period after enrollment, the 18-month period after enrollment, between 6 and 18 months, and all patient factors, including demographics (eg, education, income), self-reported health status, PAM score, and S-TOFHLA score. Statistical significance was set at the alpha=0.05 level. All analyses were completed using Stata 14.0 [25].

## Results

A total of 270 participants were enrolled from December 13, 2010 to January 24, 2012 and completed baseline surveys. Portal usage was followed for 18 months after the date of consent, from mid-June 2013 through the end of July 2013. A total of 230 participants (230/270, 85.2%) completed follow-up surveys. VA enterprise-level MHV log-in data on all participants for the full 18-month time frame became available to the investigators in 2015.

The study cohort was comprised mostly of men (228/269, 84.8%) who were white (223/270, 82.6%) and over the age of 50 years (184/270, 68.1%; Multimedia Appendix 1). Representation from women (41/269, 15.2%) was somewhat greater than the VA population of approximately 12% [26]. Fewer than 1 in 5 (46/258, 17.8%) participants had a high school education or less, approximately half completed some college (127/256, 49.2%), and one-third were college graduates (85/258, 32.9%). Health literacy screening found 98.1% (261/266) of participants in the adequate category. A total of 48.7% (128/263) of participants stated their health status as fair or poor, with only 15.0% (40/266) reporting not having a chronic condition or disability; 38.7% (103/266) resided more than a one-hour drive from the nearest VA facility.

Almost all study participants (260/268, 97.0%) reported going online at least occasionally, most commonly at home (248/268, 92.5%). A total of 32.3% (86/266) rated their computer ability as intermediate and 57.5% (153/266) rated it as advanced. Just over half (144/266, 54.1%) of the respondents indicated that they had used public Wi-Fi at an airport, coffee shop, or restaurant, while 41.9% (111/265) went online using a mobile device such as an iPhone or other mobile phone or tablet. Most respondents indicated that they had searched online to find information (256/269, 95.2%), to map a specific location (254/268, 94.8%), to shop or purchase a product (219/269, 81.4%), and for banking to pay bills (203/267, 76.0%).

**Figure 1:**
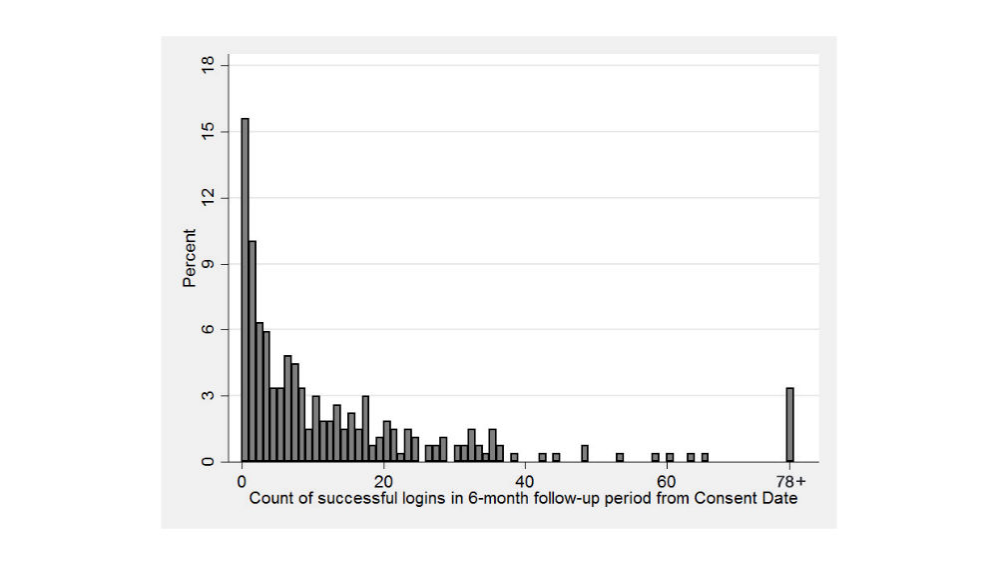
Distribution of the number of patient logins 6 months following full portal access. Total logins are capped, with participants having 78 or more logins shown at the highest count.

**Figure 2:**
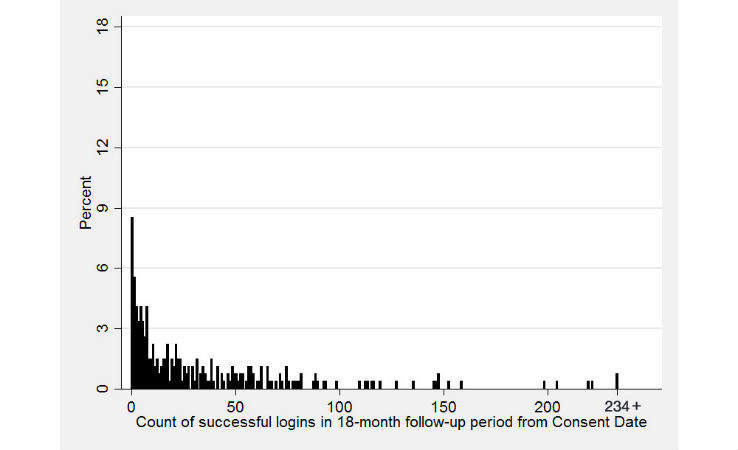
Distribution of the number of patient logins 18 months following full portal access. Total logins are capped, with participants having 234 or more logins shown at the highest count.

Short-term use of the portal was examined by analyzing the number of successful log-ins for each study participant in the 6 months following study enrollment. Long-term usage examined the total portal log-ins over 18 months. Log-ins were also measured during the time interval between 6 and 18 months following study enrollment. The distribution of log-ins for each time period is shown in Figure 1 and Figure 2, respectively (total log-ins are capped at 78 for 6-months, and at 234 for 18-months). At 6 months, the mean number of log-ins was 14.1 (standard deviation [SD] 22.7), with a median of 7, a minimum of 0, and a maximum of 169 log-ins; 75.9% (205/270) of participants had 17 or fewer log-ins. At 18 months, the mean number of log-ins was 34.8 (SD 48.7), with a median of 17, a minimum of 0, and a maximum of 407 log-ins; 75.2% (203/270) of participants had 49 or fewer log-ins.

Portal usage at 6 months, as measured by successful log-ins, is shown in Table 1. Log-in count is shown in four categories: 0 or 1 log-ins; 2 to 5 log-ins, or approximately less than once per month; 6 to 11 log-ins, or once/twice per month; and 12 or more log-ins, or over twice per month. Six months after having full access to all portal functions, approximately one-third of patients logged in less than once per month, and half logged in approximately monthly or more. Just under 16% (43/270) of patients had never logged in over 6 months.

Six-month portal usage demonstrated some variation across demographic and health-related subgroups; however, there were no statistically significant differences in log-in behavior by age, gender, education level, marital status, race/ethnicity, or distance to a VA facility. Likewise, self-reported health status, having a specific chronic condition, smoking status, or previously obtaining copies of health records were not significantly associated with variation in usage. The baseline PAM was not predictive of future short-term patient portal usage.

Table 2 shows the relationship between portal usage and participants’ reports of their technology access and Internet-related factors. Expected differences were found in the use of the portal, with significantly greater usage among those having Internet at home, having a high-speed broadband connection, and greater self-reported ability to use the Internet. The 42.5% (113/266) of participants who reported novice or intermediate abilities using the Internet used the portal less often compared to subjects reporting advanced Internet skills. More frequent log-ins were observed for regular email users, and those who were online more often, or who more frequently searched for health information. Our composite *Internet Use* measure found that while 80.3% (217/270) of the cohort fell into the *high use* category, 43.4% (23/53) of participants in the low use category never logged in or did so only once.

Patient concerns about online privacy showed no significant relationship to portal usage. Higher numbers of portal log-ins were associated with learning about MHV by reading promotional materials or from recommendations by a clinician. Portal use was also higher in participants who had visited MHV prior to completing the in-person verification step for a full access account. Participants’ views of anticipated use of specific portal functions appeared to play little role in future portal usage.

Portal usage during the 18-month period is shown in Table 3. By 18 months, never-users decreased to 9%, showing a delay in first use for some patients. Similar to the 6-month results, no significant associations were found between usage and demographics, self-reported health status, or PAM score. When assessing participant self-reports of comorbid conditions, there was a trend showing higher usage associated with having more chronic conditions, although this trend was not statistically significant. Overall use of the Internet was strongly associated with long-term portal use, similar to short-term use (data not shown).

When examining portal log-in frequency during the time interval between 6 and 18 months, as shown in Table 4, the trend between chronic conditions and log-ins was no longer observed.

Log-ins during the first 6 months after enrollment were compared to portal activity during the time interval between 6 and 18 months. Shown in Table 5, patients who rarely used the portal most commonly remained in that category. Similarly, greater usage during the first 6 months led to similar log-in behavior during the later time interval.

Many participants experienced an unsuccessful log-in during the study period; this occurred when a user entered an invalid username or password. At 6 months, a total of 169 subjects (169/270, 62.6%) had at least one unsuccessful log-in with a mean of 3.5 unsuccessful log-in attempts (SD 5.9). At 18 months, 214 subjects (217/270, 79.3%) experienced at least one unsuccessful log-in with a mean of 7.6 (SD 11.2) unsuccessful attempts.

**Table 1:** Association of demographics and health factors with portal usage 6 months after full access.

Parameter		0 or 1 log-ins, n=69 (25.5%)	2-5 log-ins, n=51 (18.9%)		6-11 log-ins, n=51 (18.9%)	12+ log-ins, n=99 (36.7%)	*P*-value
**Gender (%)**							.63
	Male	60 (88.2)	41 (80.4)		42 (82.4)	85 (85.9)	
	Female	8 (11.8)	10 (19.6)		9 (17.7)	14 (14.1)	
**Age (%)**							.19
	18-40	14 (20.6)	10 (19.6)		6 (11.8)	12 (12.1)	
	41-50	14 (20.6)	9 (17.7)		6 (11.8)	14 (14.1)	
	51-60	11 (16.2)	13 (25.5)		17 (33.3)	23 (23.2)	
	61-70	24 (35.3)	16 (31.4)		16 (31.4)	47 (47.5)	
	71+	5 (7.4)	3 (5.9)		6 (11.8)	3 (3.0)	
**Race/Ethnicity (%)**							.10
	White	52 (75.4)	40 (78.4)		47 (92.2)	84 (84.9)	
	Black	6 (8.7)	0 (0.0)		2 (3.9)	3 (3.0)	
	Hispanic	2 (2.9)	3 (5.9)		0 (0.0)	4 (4.0)	
	Other/unknown	9 (13)	8 (15.7)		2 (3.9)	8 (8.1)	
**Education (%)**							.12
	HS or less	12 (19.1)	2 (4.1)		8 (16.3)	24 (24.7)	
	Some college	30 (47.6)	29 (59.2)		23 (46.9)	45 (46.4)	
	College+	21 (33.3)	18 (36.7)		18 (36.7)	28 (28.9)	
**Marital Status (%)**							.68
	Single/widowed	17 (25.0)	11 (22.5)		10 (20.0)	16 (16.7)	
	Married	31 (45.6)	27 (55.1)		25 (50.0)	57 (59.4)	
	Divorced	20 (29.4)	11 (22.5)		15 (30.0)	23 (24.0)	
**Self-Rated Health Status (%)**							.47
	Excellent	5 (7.5)	4 (8.2)		4 (8.2)	5 (5.1)	
	Good	32 (47.8)	18 (36.7)		19 (38.8)	48 (49.0)	
	Fair	25 (37.3)	24 (49)		22 (44.9)	31 (31.6)	
	Poor	5 (7.5)	3 (6.1)		4 (8.2)	14 (14.3)	
**Patient Activation Level (%)**							.14
	Level 1	9 (13.9)	1 (2.0)		8 (15.7)	23 (23.2)	
	Level 2	14 (21.5)	11 (21.6)		10 (19.6)	14 (14.1)	
	Level 3	16 (24.6)	18 (35.3)		15 (29.4)	25 (25.3)	
	Level 4	26 (40)	21 (41.2)		18 (35.3)	37 (37.4)	
**Sought Medical Records (%)**							.37
	No	28 (41.8)	21 (41.2)		19 (37.3)	29 (29.9)	
	Yes	39 (58.2)	30 (58.8)		32 (62.8)	68 (70.1)	
**Medical Comorbidities (%)**							
	Hypertension	43 (69.4)	29 (58.0)		33 (64.7)	61 (64.2)	.67
	Heart Disease/Failure	13 (23.2)	8 (18.6)		8 (18.2)	24 (27.6)	.55
	Asthma	10 (18.5)	9 (20.5)		9 (20.9)	14 (17.5)	.96
	Diabetes	13 (22.8)	13 (30.2)		10 (23.8)	30 (32.6)	.53
	Chronic Lung Disease	6 (11.1)	7 (15.9)		8 (17.8)	15 (18.1)	.72
	Long term disability	38 (61.3)	32 (65.3)		24 (54.6)	50 (58.8)	.75
**Number of Medical Comorbidities (%)**							.18
	None	13 (19.7)	10 (19.6)		5 (9.8)	12 (12.2)	
	1 or 2	34 (51.5)	22 (43.1)		35 (68.6)	52 (53.1)	
	3+	19 (28.8)	19 (37.3)		11 (21.6)	34 (34.7)	
**Smoking Status (%)**							.88
	Never	17 (25.0)	13 (25.5)		12 (25.0)	21 (21.2)	
	Former	31 (45.6)	22 (43.1)		26 (54.2)	49 (49.5)	
	Current	20 (29.4)	16 (31.4)		10 (20.8)	29 (29.3)	
**Time to nearest VA (%)**							.59
	0-30 minutes	15 (22.7)	12 (24.0)		15 (29.4)	31 (31.3)	
	31-60 minutes	21 (31.8)	22 (44.0)		16 (31.4)	31 (31.3)	
	60+ minutes	30 (45.5)	16 (32.0)		20 (39.2)	37 (37.4)	

**Table 2:** Association of Internet access and online activity with portal usage 6 months after full access.

Parameter		0 or 1 Log-ins, n=69 (25.5%)	2-5 Log-ins, n=51 (18.9%)	6-11 Log-ins, n=51 (18.9%)	12+ Log-ins, n=99 (36.7%)	*P*-value
**Where do you access the Internet (choose all; %)**						
	Home	57 (85.1)	47 (92.2)	49 (96.1)	95 (96.0)	.046
	Friend/relative’s	20 (29.9)	21 (41.2)	11 (21.6)	18 (18.2)	.017
	Work	18 (26.9)	11 (21.6)	13 (25.5)	20 (20.2)	.75
	School	8 (11.9)	11 (21.6)	5 (9.8)	9 (9.1)	.151
**Where do you access the Internet most frequently (%)**						.025
	Home	51 (76.1)	44 (86.3)	46 (90.2)	91 (91.9)	
	Other	16 (23.9)	7 (13.7)	5 (9.8)	8 (8.1)	
**Connection Speed at Home (%)**						.012
	Not sure/none	10 (14.9)	2 (3.9)	2 (3.9)	3 (3.0)	
	Dial-up	6 (9.0)	3 (5.9)	2 (3.9)	2 (2.0)	
	High-speed	51 (76.1)	46 (90.2)	47 (92.2)	94 (95.0)	
**Ability in Using Internet (%)**						.005
	Beginner	14 (20.9)	5 (10.0)	3 (5.9)	5 (5.1)	
	Intermediate	25 (37.3)	10 (20.0)	17 (33.3)	34 (34.7)	
	Advanced	28 (41.8)	35 (70.0)	31 (60.8)	59 (60.2)	
**Do/Did you use the Internet to… (%)**						
	Email yesterday	45 (70.3)	38 (79.2)	46 (92.0)	84 (89.4)	.004
	Use search yesterday	42 (62.7)	35 (70.0)	46 (90.2)	81 (81.8)	.002
	Shop online	50 (73.5)	42 (82.4)	40 (78.4)	87 (87.9)	.12
	Bank or bill pay online	46 (67.7)	39 (76.5)	41 (82.0)	77 (78.6)	.27
	Find location of place	60 (89.6)	46 (90.2)	49 (98.0)	98 (99.0)	.015
	Look for health Information	56 (82.4)	46 (90.2)	46 (90.2)	96 (97.0)	.016
	Sign-up for health alerts	12 (17.7)	19 (37.3)	15 (29.4)	36 (36.4)	.045
**Internet Use Behavior^a^(%)**						.007
	High	46 (66.7)	43 (84.3)	41 (80.4)	87 (87.9)	
	Low	23 (33.3)	8 (15.7)	10 (19.6)	12 (12.1)	
**Concern about privacy of health information online (%)**						.28
	Very	35 (51.5)	22 (43.1)	16 (31.4)	38 (38.4)	
	Somewhat	20 (29.4)	17 (33.3)	17 (33.3)	29 (29.3)	
	Not concerned	13 (19.1)	12 (23.5)	18 (35.3)	32 (32.3)	
**How did you learn about MHV (%)**						.011
	Poster/flyer	13 (18.8)	9 (17.7)	13 (25.5)	29 (29.3)	
	Doctor/nurse	11 (15.9)	22 (43.1)	12 (23.5)	27 (27.3)	
	Other^b^	45 (65.2)	20 (39.2)	26 (51.0)	43 (43.4)	
**Very interested in using MHV to… (choose all; %)**						
	Look at lab/test results	55 (80.9)	44 (86.3)	43 (86.0)	86 (87.8)	.66
	Check for mistakes	36 (52.9)	26 (52.0)	25 (49.0)	57 (57.6)	.77
	Track weight, blood pressure, etc.	41 (60.3)	39 (76.5)	29 (56.9)	73 (74.5)	.04
	Schedule appointments	49 (72.1)	40 (78.4)	37 (75.5)	79 (79.8)	.69
	Refill medication	53 (77.9)	46 (90.2)	45 (88.2)	90 (90.9)	.08
	View care reminder	53 (77.9)	41 (80.4)	38 (76.0)	82 (83.7)	.69
	Email doctor or nurse	37 (54.4)	37 (72.6)	33 (66.0)	70 (70.7)	.11
	Learn about health condition	54 (79.4)	45 (88.2)	36 (70.6)	83 (83.8)	.11
**Visited MHV website before Premium Account (%)**						.003
	No, never	42 (62.7)	22 (44.9)	21 (42.9)	30 (30.6)	
	Yes, <once/ week	12 (17.9)	16 (32.7)	14 (28.6)	28 (28.6)	
	Yes, once/week or more	13 (19.4)	11 (22.5)	14 (28.6)	40 (40.8)	

^a^ High Internet Use Behavior represents doing 7 of 11 online activities, including: accessing the Internet; email; general search; health search; shopping; banking; geolocation; visiting any social network site; registration on any site; posting on any site; and using Facebook, MySpace, or LinkedIn

^b^ Category of *Other* includes Friend, Other Patient, VA Website, MHV Booth, and individual write-in.

**Table 3:** Association of demographics and health factors with portal usage 18 months after full access.

Parameter		0-2 Log-ins, n=49 (18.1%)	3-17 Log-ins, n=87 (32.2%)	18-35 Log-ins, n=46 (17.1%)	36+ Log-ins, n=88 (32.6%)	*P*-value
**Age, years (%)**						0.14
	18-40	10 (20.8)	15 (17.2)	7 (15.2)	10 (11.4)	
	41-50	9 (18.8)	19 (21.8)	5 (10.9)	10 (11.4)	
	51-60	8 (16.7)	22 (25.3)	10 (21.7)	24 (27.3)	
	61-70	17 (35.4)	26 (29.9)	18 (39.1)	42 (47.7)	
	71+	4 (8.3)	5 (5.8)	6 (13.0)	2 (2.3)	
**Self-Rated Health (%)**						0.24
	Excellent	6 (12.8)	4 (4.7)	5 (11.4)	3 (3.5)	
	Good	21 (44.7)	33 (38.4)	20 (45.5)	43 (50.0)	
	Fair	16 (34.0)	42 (48.8)	15 (34.1)	29 (33.7)	
	Poor	4 (8.5)	7 (8.1)	4 (9.1)	11 (12.8)	
**Medical Comorbidities (%)**						
	Hypertension	36 (73.5)	43 (49.4)	27 (58.7)	60 (68.2)	0.017
	Heart disease	11 (22.5)	10 (11.5)	13 (28.3)	19 (21.6)	0.10
	Asthma	6 (12.2)	18 (20.7)	4 (8.7)	14 (15.9)	0.28
	Diabetes	11 (22.5)	18 (20.7)	9 (19.6)	28 (31.8)	0.27
	Lung disease	5 (10.2)	8 (9.2)	9 (19.6)	14 (15.9)	0.29
	Long term disability	30 (61.2)	49 (56.3)	23 (50.0)	42 (47.7)	0.41
**Number of Health Conditions (%)**						0.07
	None	4 (8.5)	21 (24.7)	5 (10.9)	10 (11.4)	
	1 or 2	29 (61.7)	39 (45.9)	29 (63.0)	46 (52.3)	
	3+	14 (29.8)	25 (29.4)	12 (26.1)	32 (36.4)	
**Patient Activation Level (%)**						0.15
	Level 1	4 (8.7)	9 (10.5)	8 (17.4)	20 (22.7)	
	Level 2	8 (17.4)	17 (19.8)	10 (21.7)	14 (15.9)	
	Level 3	15 (32.6)	29 (33.7)	6 (13.0)	24 (27.3)	
	Level 4	19 (41.3)	31 (36.1)	22 (47.8)	30 (34.1)	

**Table 4:** Association of demographics and health factors with portal between 6 months and 18 months after full access.

Parameter		0-2 log-ins, n=88 (32.6%)	3-11 Log-ins, n=56 (20.7%)	12-23 Log-ins, n=44 (16.3%)	24+ Log-ins, n=82 (30.4%)	*P*-value
**Age, years (%)**						0.24
	18-40	15 (17.2)	13 (23.2)	4 (9.1)	10 (12.2)	
	41-50	18 (20.7)	12 (21.4)	5 (11.4)	8 (9.8)	
	51-60	21 (24.1)	10 (17.9)	12 (27.3)	21 (25.6)	
	61-70	26 (29.9)	19 (33.9)	20 (45.5)	38 (46.3)	
	71+	7 (8.1)	2 (3.6)	3 (6.8)	5 (6.1)	
**Self-Rated Health (%)**						0.34
	Excellent	8 (9.3)	2 (3.6)	5 (11.9)	3 (3.8)	
	Good	32 (37.2)	25 (45.5)	19 (45.2)	41 (51.3)	
	Fair	40 (46.5)	20 (36.4)	14 (33.3)	28 (35.0)	
	Poor	6 (7.0)	8 (14.6)	4 (9.5)	8 (10.0)	
		8 (9.3)	2 (3.6)	5 (11.9)	3 (3.8)	
**Medical Comorbidities (%)**						
	Hypertension	52 (59.1)	29 (51.8)	30 (68.2)	55 (67.1)	0.23
	Heart disease	15 (17.1)	10 (17.9)	11 (25.0)	17 (20.7)	0.72
	Asthma	16 (18.2)	9 (16.1)	3 (6.8)	14 (17.1)	0.37
	Diabetes	20 (22.7)	9 (16.1)	10 (22.7)	27 (32.9)	0.14
	Lung disease	9 (10.2)	5 (8.9)	7 (15.9)	15 (18.3)	0.30
	Long term disability	50 (56.8)	32 (57.1)	23 (52.3)	39 (47.6)	0.60
**Number of Medical Comorbidities (%)**						0.56
	None	14 (16.5)	11 (20.0)	7 (15.9)	8 (9.8)	
	1 or 2	45 (52.9)	31 (56.4)	24 (54.6)	43 (52.4)	
	3+	26 (30.6)	13 (23.6)	13 (29.6)	31 (37.8)	

**Table 5:** Relationship of portal usage in the 6 months after enrollment and from 6 to 18 months.

		Successful log-ins group at 6 months			
		Rarely/never (0-1), n (%)	Less than once a month (2-5), n (%)	Monthly to bimonthly (6-11), n (%)	Bimonthly or more (12+), n (%)
Successful log-ins group from (6 month to 18 months)	Rarely/never (0-2)	46 (66.7)	25 (49.0)	13 (25.5)	4 (4.0)
	Less than once a month (3-11)	15 (21.7)	15 (29.4)	14 (27.5)	12 (12.1)
	Monthly to bimonthly (12-23)	6 (8.7)	8 (15.7)	12 (23.5)	18 (18.2)
	Bimonthly or more (24+)	2 (2.9)	3 (5.9)	12 (23.5)	65 (65.7)

## Discussion

Among this VA cohort who took active steps to enroll in, and gain access to, patient portal functions, short-term and long-term portal usage were significantly associated with having broadband Internet at home, high self-rated ability to use the Internet, and overall online behavior. Access to broadband Internet has emerged as a social determinant of health [27], defined as, “a condition in which people are born, grow, live, work, and age, and which shapes their health status” [28]. As virtual health care becomes more commonplace, affordable broadband Internet and devices, and digital know-how, will be needed to ensure equity in care services [29].

In 2009, Kahn et al [30] identified Internet access and digital skills as being vital for the success of PHRs, stating, “if these are not made policy priorities, PHRs risk becoming a tool that is limited to groups of people who are already linked to the Internet with high health literacy and computer skills.” Our study findings indicate that ready access to the Internet and digital skills, often referred to as *digital inclusion* [31] and broader in scope than Internet access, appears to be a social determinant for exposure to patient portal services. These results expand upon prior research showing that portal adoption is associated with digital competency and Internet access [32], with usage dependent upon user skills and user-centered design of digital tools [33,34].

We expected to find that patient activation was related to more frequent portal use, but this was not the case. Limited studies examining this relationship have produced varied findings. One study found modest increases in PAM scores in portal users compared to a control group [35]. Others found no significant change in PAM scores among patients with chronic illness who were given access to a portal [36], and no association between PAM and portal log-in frequency [37]. Given our findings correlating portal use and digital access and skills, a more complex relationship likely exists between patient activation and online behavior. Larger prospective studies capturing patients’ digital environments and behaviors could offer greater insights into a patient’s context and clinical trajectory that are certain to play a role in portal usage (eg, a new diagnosis or worsening of a health condition). It is also important to characterize *stops along the way* to sustained use of digital tools [38]. Each step on the journey of patient portal engagement presents barriers and drivers to continued use [39]. Researchers should specify their targets, as there are several, including: patients routinely given a code to establish an account (which can occur with or without Internet access); patients who activate their accounts or log in at least once to test it; and ultimately, patients with sustained portal usage.

Expanding health care to virtual channels may create greater care disparities among those without affordable and reliable access to the Internet or digital devices. A focus on mutable factors that can positively impact the reach and meaningful use of portals is essential. Marketing and promotion is important, since patients may not be aware of portals, or do not perceive their value [40]. Kaiser Permanente has made a digital strategy an operational goal, and has achieved a high rate of portal engagement and use, by routinely enrolling all members and making benefits clear [41]. Clinicians also play a key role in promoting portals and elevating their significance (eg, by offering the option of secure email and explaining its appropriate use) [42].

Once online, patients will need to easily navigate portals and be able to intuitively use the tools to meet their needs, which is a necessity for sustained usage [43-45]. As consumer trends show shifts in digital devices toward mobile options, patient-facing tools need to be device agnostic. In the United States, low-income adults in particular are increasingly accessing the Internet only via mobile devices [46]. Studies in safety net and senior populations show that many people in these groups have mobile phones, and smartphones are increasingly substituting for computers [47].

Finally, user-centered designs that optimize portal usability will amplify their use [48]. Ease of entry is critical to patient satisfaction. Even the presumably simple task of securely logging-in can challenge users. Indeed, we found that many participants experienced unsuccessful log-ins. Usability challenges have been found in the VA portal, including complex password requirements for log-in [49]. Balancing security with usability is critical; users facing difficulty logging into a portal may abandon efforts altogether.

The capability to effectively use the Internet is vital for health care, as well as for education and employment. Society’s digital revolution is evolving faster than our ability to accurately measure and demonstrate digital competence across all populations [50,51]. At a minimum, health care and public health stakeholders should align across industry sectors, partnering for economic development and investment to improve community broadband and digital literacy, in an effort to achieve digital inclusion.

### Limitations

There are limitations to our study. Patients were recruited at a single VA facility, and may not represent a general population of patients or those enrolling for the portal. Participants may be more computer-savvy than the general portal user population. Many participants visited the MHV website before the study (VA patients who register but do not complete identify proofing can refill medications using a prescription number). The study setting in a busy MHV office precluded capturing data on all patients informed about the study. However, such issues could potentially underestimate challenges that users faced using the portal. Second, measuring the portal served as a proxy for usage and benefit. While standardized metrics for capturing patient usage of digital tools have not been established, common measures include initial enrollment, log-in frequency, and using specific functions [34]. Measuring total log-ins during 6-month and 18-month intervals is not ideal; however, repeated log-ins over time signals user value. Third, our health literacy assessment found virtually all patients at the highest level of the S-TOFHLA; the acceptability of this instrument has since been questioned [52]. Finally, study subjects may have experienced inconsistent connections to the Internet over the study, complicating the measurements of associations between digital inclusion and portal use.

### Conclusion

The ultimate impact of patient portals will rest on their ability to reach across populations and have real-world effects on self-care and outcomes. Realizing potential benefits will require not just initial adoption but also sustained portal usage. Strategies and novel methods to enhance comfort with digital devices and increase Internet skills, along with affordable access to broadband and wireless connections, are required ingredients as the health care community offers an increasing array of online tools and services. There are important relationships between digital inclusion and the use and benefits of virtual care tools.

## Appendix

Multimedia Appendix 1 Study cohort self-reported demographics, conditions, patient activation, and Internet access.

## Abbreviations

MHV: My HealtheVet
PAM: Patient Activation Measure
SD: standard deviation
S-TOFHLA: Short Form Functional Health Literacy Assessment
VA: Veterans Affairs
