# Supplementary material for: The Association of Patient Factors, Digital Access, and Online Behavior on Sustained Patient Portal Use: A Prospective Cohort of Enrolled Users
Source: J Med Internet Res. 2017 Oct 17;19(10):e345. doi: 10.2196/jmir.7895 (PMC5663951; doi:10.2196/jmir.7895)
Supplement: Multimedia Appendix 1 [file jmir_v19i10e345_app1.pdf]

Multimedia Appendix 1: Study cohort self-reported demographics, conditions, patient activation, and Internet access.

	Total, N=270 (%)
Gender	
Male	228 (84.8)
Female	41 (15.2)
Age	
18-40	42 (15.6)
41-50	43 (16.0)
51-60	64 (23.8)
61-70	103 (38.3)
71+	17 (6.3)
Race/Ethnicity	
White	223 (82.6)
Black	11 (4.1)
Hispanic	9 (3.3)
Other/Unknown	27 (10.0)
Education	
High school or less	46 (17.8)
Some college	127 (49.2)

College+	85 (33.0)
Marital Status	
Single/Widowed	54 (20.5)
Married	140 (53.2)
Divorced	69 (26.2)
Health Status	
Excellent	18 (6.8)
Good	117 (44.5)
Fair	102 (38.8)
Poor	26 (9.9)
Self-Reported Condition	
Hypertension	166 (64.3)
Long term disability	144 (60.0)
Diabetes	66 (28.2)
Heart Disease/Failure	53 (23.0)
Asthma	42 (19.0)
Chronic Lung Disease	36 (15.9)
Number of Medical Comorbidities	
None	40 (15)

1 or 2	143 (53.8)
3+	83 (31.2)
Smoking Status	
Never	63 (23.7)
Former	128 (48.1)
Current	75 (28.2)
Patient Activation Level	
Level 1	41 (15.4)
Level 2	49 (18.4)
Level 3	74 (27.8)
Level 4	102 (38.4)
Time to nearest VA	
0-30 minutes	73 (27.4)
31-60 minutes	90 (33.8)
60+ minutes	103 (38.7)
Where Internet Accessed	
Home	248 (92.5)
Friend/Relative’s	70 (26.1)
Work	62 (23.1)
School	33 (12.3)
Internet Speed (Home)	
Not Sure/None	17 (6.3)

Dial-up	13 (4.9)
High-speed	238 (88.8)
Self-Rated Internet Ability	
Beginner	27 (10.2)
Intermediate	86 (32.3)
Advanced	153 (57.5)
